# Retrospective cohort study on radial head arthroplasty comparing long-term outcomes between valgus type injury and fracture dislocation

**DOI:** 10.1186/s12891-020-03767-4

**Published:** 2020-11-20

**Authors:** Alvin Chao-Yu Chen, Chun-Jui Weng, Chih-Hao Chiu, Shih-Sheng Chang, Chun-Ying Cheng, Yi-Sheng Chan

**Affiliations:** Bone and Joint Research Center, Department of Orthopaedic Surgery, Chang Gung Memorial Hospital–Linkou and Chang Gung University College of Medicine, 5th, Fu-Shin Street, Kweishan District, Taoyuan, 333 Taiwan, ROC

**Keywords:** Radial head arthroplasty (RHA), Loose fit, Valgus injury, Fracture dislocation, Osteolysis, Correlation analysis

## Abstract

**Background:**

Radial head arthroplasty (RHA) has been commonly adopted for irreparable radial head fractures while little information is addressed on valgus type injury. The purpose of this study is to report long-term outcomes and radiographic analysis in RHA for valgus type injury with comparison to fracture dislocation injury.

**Methods:**

A retrospective cohort study was conducted in patients receiving unilateral RHA with loose-fit, modular metal prosthesis for irreparable radial head fractures between 2004 and 2012. Totally, 33 patients with a mean follow up of 9 years (range, 7 to 15 years) were enrolled and divided into two groups including 14 valgus injuries and 19 fracture-dislocations. Demographics of the patients, injury details, clinical and radiographic outcomes, and correlation analysis were investigated and compared between two groups.

**Results:**

In patient demographics, significant difference was noted in sex distribution (*p* = 0.001), lateral collateral ligament involvement (*p* = 0.000) and time from injury to RHA (*p* = 0.031) between two groups. No patient underwent subsequent removal or revision of prosthesis. Good to excellent results according to Mayo Elbow Performance Score (MEPS) was achieved in 13 and 14 patients in group A and B respectively. Final motion range and Disabilities of the Arm, Shoulder, and Hand score was significantly better in valgus injury group. Radiographic analysis demonstrated fewer patients in valgus injury group presented periprosthetic osteolysis with weak to moderate negative correlation between radiolucency score and MEPS.

**Conclusions:**

With an average of 9 years follow-up, RHA using loose-fit, modular metal prosthesis achieves encouraging outcomes for both valgus injury and fracture dislocation. In valgus type injury, better motion range, lower disability score and lower incidence of periprosthetic osteolysis is noted while correlation analysis of radiolucency score suggests extended, long-term investigation.

## Background

Given that it has long been debated about rationales of prosthesis replacement in the elbow fractures involving radial head [1], radial head arthroplasty (RHA) is generally considered a feasible alternative to radial head resection for irreparable radial head fractures [2]. Since up to 30% of radial head fractures are associated with ligamentous injuries [3], meticulous management of both osseous and soft tissues is critically related to functional outcome. With increasing recognition of radial head as an important stabilizer [4, 5], restoration of radiocapitellar alignment to facilitate surrounding tissue healing is popularly adopted and becomes the main goal in treatment of radial head fractures [6, 7]. Several kinds of prostheses have been available for radial head replacement [8] with a growing number of articles showing advancements in implant designs and favorable outcome. However, prosthetic RHA has yet to be the gold standard for irreparable radial head fractures basically owing to insufficiency in long-term reports [9] and lack of outcome correlation in ligamentous injuries [10, 11] in the majority of clinical reports. In addition, most of current articles concerning outcome assessment and complication analyses are mainly focused on unstable fractures with elbow dislocation and concomitant lateral ligament injury [12]. RHA in traumatic valgus instability has less been fully addressed. This led us to perform a retrospective, case-control study, and hypothesize that RHA in valgus type injuries yielded different functional and radiographic outcomes. The primary aim of this study is to investigate the patient characteristics and functional outcomes of RHA for irreparable radial head fractures in traumatic valgus type injuries through a retrospective cohort review. The secondary aim is to compare the clinical and radiological differences of RHA between valgus type injury and fracture dislocation type injury.

## Methods

### Cohort groups

Based on a retrospective cohort study between 2004 and 2012, unilateral RHA using a modular smooth-stemmed radial head implant (Evolve; Wright Medical Technology) was identified in 49 patients with comminuted radial head fractures (Mason type III and IV). Patients with less than 7-year follow up, other ipsilateral arm fracture, Essex Lopresti injury, insufficient medical record, and poor radiographic quality were excluded. A total of 33 patients were enrolled in this report. All the surgeries were approved preoperatively by the audit committee in our department with surgical indication well documented in the medical records. There were 13 female and 20 male patients with an average age of 44.76 ± 13.25 years (range, 24 to 75 years). RHA was the primary surgery after injury in 25 patients and, revision surgery after previous fixation failure (from 1 to 3 surgeries) in 8 patients. All had regular follow-up for more than 2 years postoperatively. The latest survey was at an average of 9.03 ± 1.74 years (range, 7 to 15 years). The 33 patients were divided into two groups based on the type of instability. Group A was valgus-type injury and consisted of 14 patients. Group B was radial head fracture dislocation in 19 patients.

### Surgical procedure

All surgery was performed in general anesthesia and supine position by one single surgeon. Radial head fracture was explored with lateral Kocher approach. All patients underwent RHA with an uncemented modular prosthesis (EVOLVE radial head system, Wright Medical Group, Arlington, TN), which included a head segment and a smooth stem with options for head thickness and neck length. The size of the head segment was 1 to 2-mm downsized by reassembling the major fragments of the radial head on a sizing tray. The stem diameter was determined after sequential reaming of radius canal and calcar trimming. The final height of implanted prosthesis was adjusted by the combination of proper head thickness and neck length and was set with the proximal margin to reach the horizontal level of coronoid tip, which was confirmed by the mini c-arm image intensifier during surgery.

Lateral ligament-capsular structure was torn in 16 patients, and reattached using Mitek GII anchor (Mitek Surgical Product or Twinfix Ti anchor (Smith & Nephew Endoscopy, Andover, MA) suture fixation and No.2 ethibond (Ethicon, Somerville, NJ, USA) suture augmentation after completion of radial head replacement. Medial collateral ligament was explored and fixed with suture anchor repair in three patients (two in group A and 1 in group B) who presented grade III or more instability on valgus test after radial head prosthesis implantation.

### Clinical evaluation

Study approval from Institutional Review Board (IRB 201800206B0) was obtained for patient data retrieval and invitation to patients in returning for clinical evaluation. Implant data of radial head prostheses were located through the National Health Insurance Administration Register, which contain original registration files and claim records for reimbursement. Clinical data review and collection was performed by one of the co-authors, who was blinded to the patients’ individual files. Functional survey was performed using the Mayo Elbow Performance Score (MEPS) and shortened Disabilities of the Arm, Shoulder, and Hand (QuickDASH) score. Residual pain around the involved elbow was recorded in the visual analog scale (VAS), ranging from 0 to 10.

### Radiographic investigation

**Fig. 1:**
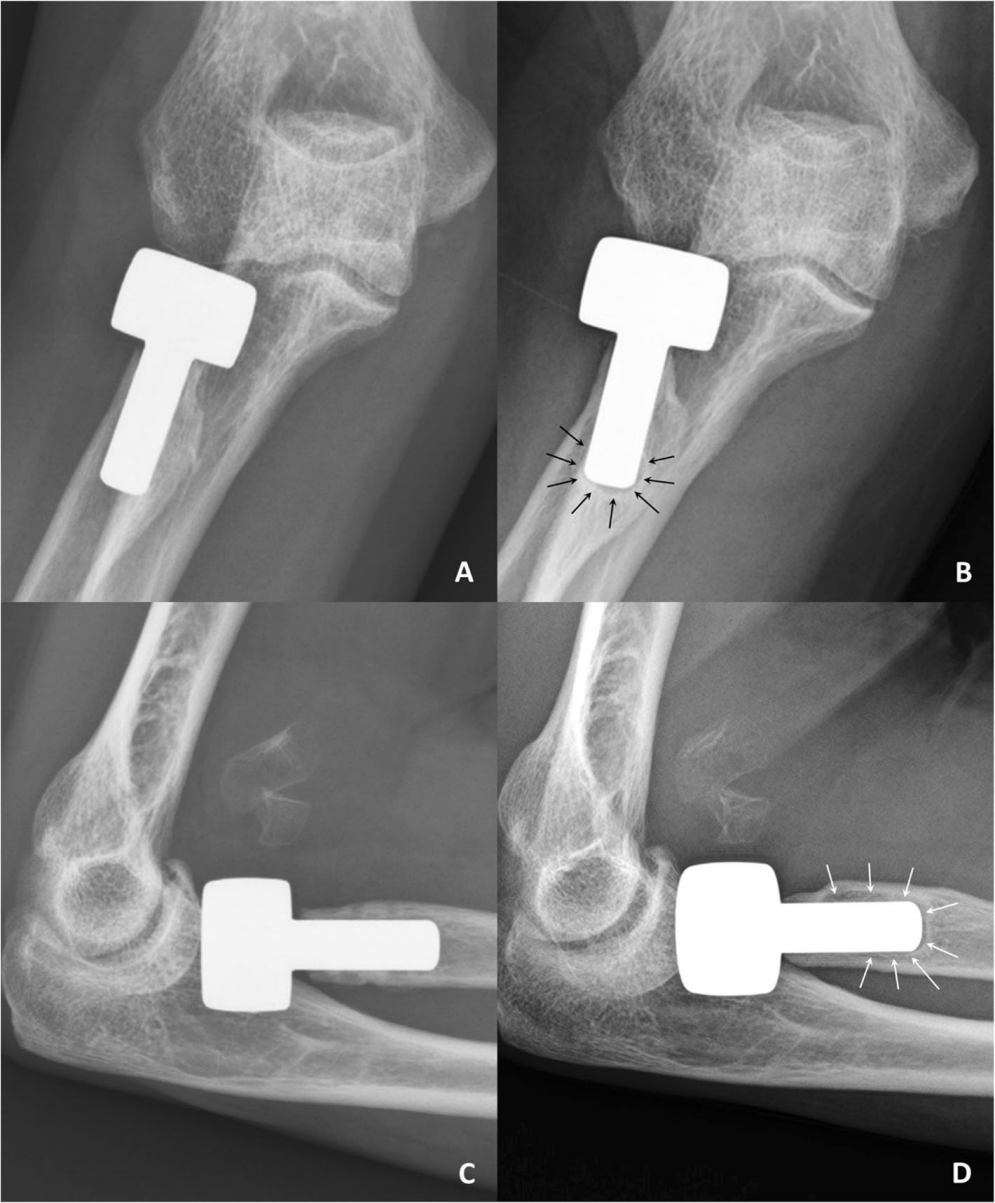
Right elbow radiographs from a 54 year-old female patient after radial head arthroplasty. **a** Anteroposterior view at 3 months. **b** Anteroposterior at 11 years showing periprosthetic osteolysis (black arrows). **c** Lateral view at 3 months. **d** Lateral view at 11 years showing periprosthetic osteolysis (white arrows)

**Fig. 2:**
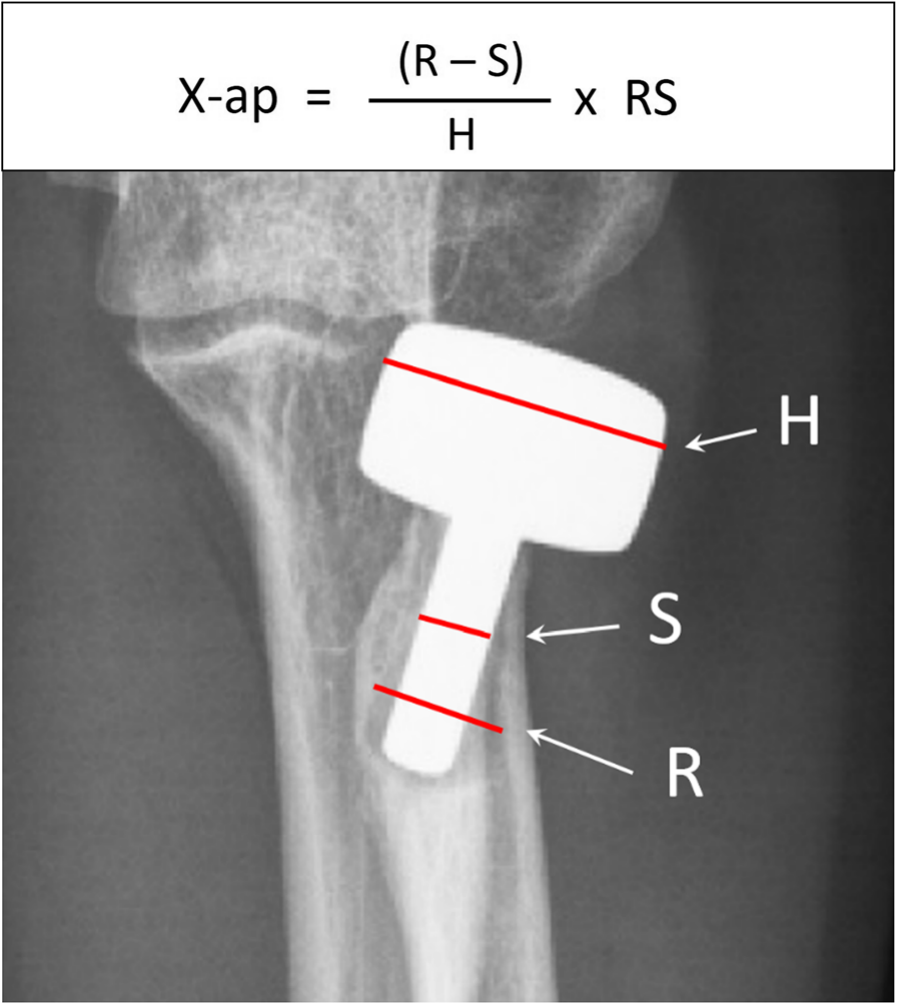
Measurement of periprosthetic radiolucency on anteroposterior view (X-ap) by 3 red lines on radiographs. H red line: width of radial head component on radiographs. S red line: width of stem on radiographs. R red line: maximal width of radiolucency on radiographs. RS: real size in head component of the prosthesis

Radiographs of anteroposterior (AP) and lateral projections were performed for each elbow. AP view was taken with forearm in maximal supination and elbow in maximal extension; lateral view, with forearm in neutral rotation and elbow in 90° flexion. Two of co-authors performed radiographic evaluation including radiolucency around the prosthesis stem, presence of osteoarthrosis, and heterotopic ossification. High resolution images of AP and lateral views were meticulously compared with postoperative radiographs to identify the location of periprosthetic osteolysis (Fig. 1). Evaluation of periprosthetic osteolysis on radiographs was performed by drawing a line across the stem at the location with maximal width of radiolucency, which was then calibrated with head and stem size (Fig. 2). The sum of maximal width of radiolucency in AP and lateral views was recorded for each measurement. Radiolucency score of each elbow was defined as the average of measurement with two evaluators [13].

### Statistical analysis

Descriptive statistics were calculated for analysis of the key variables with comparison between two groups. In the primary outcome survey, a chi-square test was used for calculating categorical data (sex, injured side, injured hand and complication rate); an independent t-test, for normally distributed data (patient age, time from injury to surgery and surgical times). For the secondary outcome measurement, the Mann-Whitney rank sum test was used in comparing the data that were not normally distributed (VAS, motion range, radiolucency sore, MEPS, and QuickDASH). A *p*-value of < 0.05 was considered statistically significant. Pearson correlation was used to explore the correlation between radiolucency and functional scoring.

## Results

Based on the analyses of patients characteristics (Table 1), there was significant difference in sex distribution (*p* = 0.001), lateral ligament involvement (*p* = 0.000) and time from injury to RHA (*p* = 0.031). There was a trend of younger age in fracture dislocation group, but the difference was insignificant (*p* = 0.052). There was equal number of patients receiving RHA after failed fixation surgery between two groups; no significant difference was found (*p* = 0.316). One in group A had failed to percutaneous pinning; three in group A and four in group B had failed to plate fixation. Other items including dominant hand involvement, concomitant medial collateral ligament repair and prior surgery all presented no significant difference. None of the patients in both groups underwent or needed subsequent removal of the radial head prosthesis at the latest visit.

**Table 1:** Patient Characteristics

Outcome survey	Group A ***N*** = 14	Group B ***N*** = 19	***P***-value
Age (years)	49.93 ± 13.3	40.95 ± 12.16	.052
Sex			.001*
Men	4 (29%)	16 (84%)	
Women	10 (71%)	3 (16%)	
Dominant side injured	9 (64%)	9 (47%)	.174
Right arm	8 (57%)	10 (53%)	
Left arm	6 (43%)	9 (47%)	
Ligament repair			
Lateral collateral ligament	0	16 (84%)	.000*
Medial collateral ligament	2 (14%)	1 (5%)	.194
Time from injury to replacement (months)	6.14 ± 7.72	2.03 ± 4.4	.031*
Radial head replacement			
As a primary surgery	10 (71%)	15 (79%)	.316
As a revision surgery	4 (29%)^a^	4 (21%)	.316

^a^ revision surgery after failed surgical fixation

* A *p*-value of < 0.05 indicated significant difference

### Functional outcome

There were 13 patients (93%) in group A and 14 patients (74%) in group B were rated good to excellent according to MEPS. One patient in group A and 4 patients in group B was rated; only one patient in group B rated poor. Clinical results were compared between two groups (Table 2). There were totally 25 patients (76%) presenting residual pain or soreness of the injured elbow; 11 patients (79%) were in group A and 14 patients (74%), in group B. All except one were mild pain (VAS = 1 to 3). One patient in Group B presented moderate pain (VAS = 5) on regular activity; radiographs showed posttraumatic arthrosis of ulnohumeral articulation. With regard to the functional survey, significant difference was noted in motion range and QuickDASH score. Averaged motion arc was greater in group A (range, 110° to 140°) than in group B (range, 65° to 140°) with a *p*-value of 0.034. Better functional score based on QuickDASH was found in group A (range, 0 to 18.2) than in group B (range, 0 to 43.2) with a *p*-value of 0.046. However, functional survey according to MEPS showed no significant difference between group A (range, 70 to 100) and group B (range, 45 to 100). There was no infection or major complication in both groups. Late complications including two cases of ulnohumeral arthrosis, three cases of heterotopic ossification, one case of elbow stiffness with a motion range less than 90 degrees, and one revision surgery for contracture release. No significant difference regarding complication rate was noted between two groups.

**Table 2:** Functional outcome

Outcome survey	Group A N = 14	Group B N = 19	***P***-value
Latest survey (years)	9.14 ± 2.28	8.95 ± 1.27	.378
VAS	1 ± 0.78	1.42 ± 1.26	.140
Range of motion (degree)	126.79 ± 7.99	114.47 ± 19.5	.034*
MEPS	87.5 ± 10.13	80.53 ± 15.27	.152
QuickDASH score	6.17 ± 7.33	15.43 ± 15.41	.046*
Complication	3 (21%)	4 (21%)	.490
Ulnohumeral arthrosis	1 (7%)	1 (5%)	
Heterotopic ossification	2 (14%)	1 (5%)	
Stiffness (< 90° motion range)	0	1 (5%)	
Second surgery	0	1 (5%)	

*VAS* Visual analog scale

MEPS Mayo Elbow Performance Score

*QuickDASH score* Shortened Disabilities of the Arm, Shoulder, and Hand score

* A *p*-value of < 0.05 indicated significant difference

### Radiographic analysis

Periprosthetic osteolysis was meticulously evaluated on the latest radiographs, which were all blinded to two evaluators. There were totally 26 patients presenting periprosthetic osteolysis (Table 3); 9 were in group A (64%) and 17, in group B (89%); the difference in positive incidence between two groups was significant (*p* = 0.043). Regarding radiolucency scores, there was no significant difference between group A (range, 0 to 4.15) and group B (range, 0 to 8.74). Pearson correlation coefficient (PCC) was used to investigate the correlation between radiolucency score and functional scores. MEPS exhibits low to moderate negative correlation with radiolucency score in group A (PCC = − 0.384), but not in group B (PCC = − 0.035). No remarkable correlation was found between radiolucency score and other functional scores including VAS and QuickDASH between two groups.

**Table 3:** Radiographic outcome

Radiographic analysis	Group A N = 14	Group B N = 19	***P***-value
Patients with radiolucency	9 (64%)	17 (89%)	.043*
Radiolucency score	2.14 ± 2.47	2.92 ± 2.41	.369
Correlation^a^ with VAS	**­** .049	.130	
Correlation with MEPS	**- .384**^b^	**-** .035	
Correlation with QuickDASH	.159	.010	

*VAS* Visual analog scale

*MEPS* Mayo Elbow Performance Score

*QuickDASH score* Shortened Disabilities of the Arm, Shoulder, and Hand score

^a^ Pearson correlation coefficient (PCC)

^b^ PCC between −0.3 to −0.5 shows weak to moderate negative correlation

* A *p*-value of < 0.05 indicated significant difference

## Discussion

The strength of this report is a single-unit cohort study on a long-term base. Both validated clinical assessment and objective radiographic survey are performed with a comparison between two different types of injuries after RHA. Our study demonstrates encouraging results of RHA for irreparable radial head fractures at an average of nine-year follow up in both valgus injury cases and fracture dislocation cases. None receive subsequent removal or revision of radial head prosthesis in both groups. With further comparison of patient characteristics between two groups, statistically significant difference is noted in sex distribution, lateral collateral ligament involvement and time to RHA. While recent studies revealed higher incidence of radial head fractures in women with older age, there was scarce evidence to document significant correlation to surgical outcomes [14]. Nevertheless, all those differences in patient characteristics may exert considerable influence in functional and radiographic outcomes and warrant further investigation and comparison between two types of patients on a long-term base.

Biomechanical studies anecdotally analyzed stabilizing influence of radial head replacement based on valgus type injuries with medial collateral ligament insufficiency [15, 16]. However, little clinical information has been addressed on the long-term outcomes after RHA in irreparable radial head fractures with traumatic valgus instability. Harrington IJ, et al. reported early experience on RHA between 1966 and 1979; eligible criteria was radial head fractures combined with elbow dislocation, fracture of the proximal ulna, fracture of a major portion of the coronoid process and the medial ligament tear [17]. Subsequent publication with a mean follow-up of 12 years confirmed the long-term function of RHA including two patients of valgus type injury [18]. Another publication in mid- to long-term results after bipolar RHA also reported excellent to good outcome while with a heterogeneity of injury patterns [19].

In the index study, surgical outcomes of RHA is analyzed and compared between valgus injury group and fracture dislocation group at an average of postoperative 9 years. Significantly better results regarding elbow motion range and QuickDASH scores are found in valgus injury group than in fracture-dislocation group. While higher MEPS score and lower VAS are also found in valgus injury group, the difference is insignificant. This could be partially attributed to late treatment in more patients of valgus injury group. Based on comparison of patient characteristics between two groups, significantly longer time interval from injury to RHA is found in valgus injury group than in fracture dislocation group. There is a trend for the patients in valgus injury group to have treatment delay, which could be a negative influence and lead to less remarkable difference in MEPS. Postoperative complication is comparable in both groups, and not directly related to the prosthesis implantation. Therefore, early decision making and selection of proper candidates for RHA is recommended in optimization of treatment outcomes.

Late complications with RHA are commonly reported in the literature including arthrosis, pain, elbow stiffness, and heterotopic ossification. In our cases, no significant difference is noted regarding overall complication between two groups. There is an equal patient number of arthrosis in each group, and none of the radiographic analysis presented capitellar erosion. Literature review showing capitellar erosion and arthrosis has been a general concern following RHA [20, 21]. Recent biomechanical studies comparing different prosthesis design suggest greater radiocapitellar contract in monoblock prosthesis may better resist instability and lessen cartilage attrition [22, 23]. While the reported outcome favors monoblock design in our series as well as other clinical studies [8], avoidance of cartilage erosion and arthrosis could be attributed to many factors and technical demands in addition to prosthesis profiles [24].

Periprosthetic osteolysis has been commonly observed around smooth-stemmed radial head prosthesis with loose-fit implantation. In our study, the incidence is significantly higher with fracture dislocation group than valgus injury group. By measuring the osteolysis area from latest radiographs, radiolucency score is also higher in the fracture dislocation group while the difference is not significant. PCC reveals no correlation is found in radiolucency score with VAS and QuickDASH in both groups, and with MEPS in fracture dislocation group. This is comparable with short- to mid-term reports in previous publication [25, 26]. However, analysis between radiolucency score and MEPS in valgus injury group exhibits low to moderate correlation, which warrants extended cohort study with long-term survey.

Several limitations in our study include relatively small sample size with heterogeneity of trauma severity. Pre-injured status of the involved limb could not be fully assessed owing to the retrospective nature. Finally, the missing data in the cases excluded due to insufficient medical records may exert a possible influence on the statistical outcomes.

## Conclusion

Modular metal prosthesis with loose-fit implantation for irreparable radial head fractures exhibits encouraging outcomes both in cases of valgus injury and fracture dislocation at an average of 9 years follow-up. In valgus injury group, clinical survey shows better motion range, lower QuickDASH scores and lower incidence of periprosthetic osteolysis while correlation analysis of radiolucency score may imply a sustained influence and warrant extended investigation on a long-term base.

## Data Availability

The datasets generated during the current study are available from the corresponding author on reasonable request.

## Abbreviations

RHA: Radial head arthroplasty
MEPS: Mayo Elbow Performance Score
QuickDASH: Disabilities of the Arm, Shoulder, and Hand
VAS: Visual analog scale
AP: Anteroposterior
PCC: Pearson correlation coefficient
